# Supplementary material for: Development of an intervention for reducing infant bathing frequency
Source: PLoS One. 2024 Feb 29;19(2):e0298335. doi: 10.1371/journal.pone.0298335 (PMC10903808; doi:10.1371/journal.pone.0298335)
Supplement: S1 Table — (DOCX) [file pone.0298335.s001.docx]

### Supporting Material S1: Table S1: Consolidated criteria for reporting qualitative research (COREQ) items

| **Domain 1: Research team and reflexivity** | |
| --- | --- |
| *Personal Characteristics* | |
| 1. Interviewer/facilitator | Author LG conducted the phase 1 interviews. Authors LG, MU and MRP conducted the stage 3 workshop and stage 4 study walkthrough. |
| 2-5; Personal characteristics of researchers (credentials, occupation, gender, experience and training). | • Dr Lucy P. Goldsmith PhD BSc FHEA Postdoctoral Research Fellow, experienced in both qualitative and quantitative methods, trained in qualitative research, experienced in intervention development. Female.  • Dr Michael R. Perkin, PhD MB BS (Hons) Reader in Clinical Epidemiology and Consultant in Paediatric Allergy. Male.  • Dr Robert J. Boyle, PhD MB ChB MRCP (Paed). Consultant paediatric allergist, Clinical Reader in Paediatric Allergy and Director of a Paediatric Research Unit. Male.  • Prof Carsten Flohr BM BCh MA PhD FRCP FRCPCH Professor of Dermatology, male, Fellow of the Royal College of Physicians and the Royal College of Paediatrics and Child Health. Chair in Dermatology and Population Health Science and director of the Unit for Population-Based Dermatology Research. Consultant in Dermatology and Research & Development Lead in the NHS.  • Amanda Roberts, Expert patient and patient carer representative. Member of the Centre for Evidence Based Dermatology and Nottingham Support Group for Carers of Children with Eczema. Female.  • Dr Lakshmi Chandrasekaran, MSc, MBBS, BSc. Academic Clinical Fellow in General Practice, female, trained in qualitative research methodology.  • Dr Charlotte Wahlich DPsych, MSc, BSc. Health Psychologist and Postdoctoral Research Fellow, female, experienced in qualitative research methods and intervention development. Trained in qualitative research.  • Kathryn Willis, MSc, BSc, Research Assistant, Female, experienced in qualitative methods and intervention delivery.  • Prof Michael Ussher PhD MSc BA Professor of Behavioural Medicine, experienced in qualitative methods, trained in qualitative research, health psychologist, experienced in intervention development. Male. |
| 6. Relationship established | Stages 1 and 4: No participants formally recruited to the study were known to researchers prior to the commencement of the study.  Stage 3: Purposively sampling was used for recruitment including contacts and recommended contacts of colleagues. Both PPI experts were known to at least one of the researchers. |
| 7. Participant knowledge of the interviewer | Stages 1 and 4: Participants knew that the interviewer was a researcher conducting the interviews as part of a larger project, working alongside a team of researchers.  Stage 3: Participants included professional colleagues with knowledge of the research interests of the team. |
| 8. Interviewer characteristics | See ‘reflexive note’ |
| **Domain 2: study design** | |
| *Theoretical framework* | |
| 9. Methodological orientation and theory | Our epistemological stance in this study was critical realism – which assumes that experiences are understood through human interpretation and mediated by our beliefs and perceptions. We consider how the authors’ beliefs and experiences may have affected the research process in the reflexive note. We analysed the data using thematic analysis. |
| 10. Sampling | Stages 1 and 4: Quota sampling was used, attempting to fulfil the sampling framework.  Stage 3: Purposive sampling was used to obtain representation from all relevant specialist disciplines. |
| 11. Method of approach | See text |
| 12. Sample size | Phase 1 n=31; Phase 3 n=13; Phase 4 n=5 |
| 13. Non-participation | Stage 1: n=25 refused to participate or dropped out; reasons included: giving birth before the interview was arranged: (n=3), losing interest in the study (n=2), feeling like they didn’t have enough to say about bathing babies (n=2) or not responding to contacts about the study (n=18)  Stage 3: n=3 refused to participate or dropped out; reasons included: prior commitment on the date of the workshop (n=2), extended period of leave (n=1).  Stage 4: n=3 refused to participate or dropped out; reasons included finding it difficult to join the focus group (n=1), unknown reason why the participant did not join the focus group (n=2). |
| 14. Setting of data collection | Stage 1: The researcher conducted interviews alone from a private office and participants were all at home or work.  Stage 3: The stakeholder workshop was held at St George’s, University of London.  Stage 4: The PPI focus group was held online. |
| 15. Presence of non-participants | Stage 1: The researcher was alone in a private office and some participants were alone, some participants had others entering and exiting the room.  Stage 3: Only researchers and workshop participants were present.  Stage 4: All participants were at home and may have had others present during the focus group. |
| 16. Description of sample | See text |
| *Data collection* | |
| 17. Interview guide | See text and supporting material S2 |
| 18. Repeat interviews | Two participants from stage one interviews were later recruited to participate in the stage 4 PPIE study walkthrough. |
| 19. Audio/visual recording | Audio recording was used for all data collection stages. Data from stages 1 and 3 were transcribed by a professional transcriber. |
| 20. Field notes | Field notes were made during all stages of the study and used to inform the analysis. Stage 3 field notes were checked by participants. |
| 21. Duration | Stage 1: mean interview length = 43 minutes; (SD=10 minutes)  Stage 3: two hours contact time; plus additional contact via email. Stage 4: one hour contact time. |
| 22. Data saturation | Data saturation was discussed toward the end of the stage 1 and following the final stage 4 interview. In stage 3 it was discussed in the context of additional rounds of requesting comments about redrafted materials following the workshop. |
| 23. Transcripts returned | Transcriptions were not returned to participants for comment. |
| **Domain 3: Analysis and findings** | |
| *Data analysis* | |
| 24. Number of data coders | Five researchers coded the data (LG, LC, MU, CW and KW). |
| 25. Description of the coding tree | The initial version of the coding tree was developed using both deductive (theory-driven) and inductive coding. The coding tree identified themes and sub-themes focussing on potential barriers and facilitators to intervention engagement. |
| 26. Derivation of themes | Themes were both derived from theory (deductive coding) and the data (inductive coding). |
| 27. Software | NVivo 12 was used to code and manage data. |
| 28. Participant checking | Two of the twenty mothers interviewed at stage joined the study walkthrough (stage 4) alongside new mothers to provide feedback on the intervention at a late stage of the design having contributed to the design in the first phase. In addition, all stakeholder workshop participants were asked to provide comments on the redrafted materials via email. |
| *Reporting* | |
| 29. Quotations presented | Co-authors (CW, MU) verified whether the quotations illustrate the themes/findings. |
| 30. Data and findings consistent | Co-authors (CW, MU) verified that the data and findings were consistent. |
| 31. Clarity of major themes | Co-authors (CW, MU) checked that the major themes are clearly presented in the findings. |
| 32. Clarity of minor themes | Co-authors (CW, MU) checked the clarity of minor themes. |
