# Supplementary material for: Development of an intervention for reducing infant bathing frequency
Source: PLoS One. 2024 Feb 29;19(2):e0298335. doi: 10.1371/journal.pone.0298335 (PMC10903808; doi:10.1371/journal.pone.0298335)
Supplement: S1 File — (PDF) [file pone.0298335.s002.pdf]

Participant unique ID: | | | | | |

Verbal consent Form for Participants

Version 1.2 (10/08/22) IRAS project ID: 311086
Chief investigator: Dr Michael Perkin

Title of study:
Interviews exploring issues around whether families are willing to change baby bathing practice during the first months of life (BabyBathe WP-1)

Instructions for researcher: Please read out each statement below before the interview (participant will also have a copy of this form) and ask the participant to confirm they consent. A copy of the consent transcription script and the part of the recording which provides evidence of consent will be retained.

Researcher to Initial box

1.	I confirm that I have read and understood the information sheet (Version 1.2 dated 10/08/22) for the above study. I have had the opportunity to ask questions and they have been answered satisfactorily.	
2.	I understand that my participation is voluntary and that I am free to withdraw from the interview at any time, without giving any reason and without my care or legal rights being affected.	
3.	I agree to have the interview audio-recorded or video-recorded and to anonymised quotes from the interview being used when reporting the findings	
4.	I understand that all information collected about me will be strictly confidential and that some data will be collected and stored at St George's, University of London (who are overseeing the study) outside the NHS in secure, password-protected files.	
5.	I understand that relevant sections of any of my medical notes and personal data collected during the study may be looked at by authorised individuals from St George's, University of London, St George's University Hospitals NHS Foundation Trust or from regulatory authorities, where it is relevant to my taking part in this study. I give permission for these individuals to have access to my records.	
6.	I agree to take part in this study.	
	OPTIONAL:	
7.	I agree to be contacted about taking part in an additional focus group to discuss how we might best support families to change their infant bathing routines.	Yes ☐<br>No ☐

Name of Participant

____

Name of researcher taking consent

Date

Signature

_______________________________ __________________ _________________________

When completed: Researcher to keep original copy and one copy for participant
