# Supplementary material for: Development of an intervention for reducing infant bathing frequency
Source: PLoS One. 2024 Feb 29;19(2):e0298335. doi: 10.1371/journal.pone.0298335 (PMC10903808; doi:10.1371/journal.pone.0298335)
Supplement: S2 File — (DOCX) [file pone.0298335.s003.docx]

### Supporting Material S2: Topic Guide

**Topic guide Baby Bathe WP-1**

Version 1.0 (27/07/22) IRAS project ID: 311086

- Check participants are ideally:
  - planning to bathe their baby a minimum number of times per week
  - open to being flexible about this
  - open to randomisation
- Emphasise equipoise

**Aims and objectives of study**

The overall aim of WP1 is to identify potential barriers and facilitators to uptake and maintenance of an intervention to reduce bathing frequency during early infancy, among families expecting a baby.

Objectives are to:

- interview women with and without atopy in their family, and with and without children, and with a mix of ethnicities, ages and socio-economic status, to explore potential barriers and facilitators;
- analyse the interview/focus group data to produce a theoretically based description of the barriers and facilitators

**Introduction to the study**

- 1. Interviewer introduces themselves and asks interviewees to introduce themselves.

How many interviewees are there?

Give number here:

What are the roles of interviewees, regarding the baby in question?

Tick all that apply:

| Mother to be |  |
| --- | --- |
| Partner/spouse |  |
| Grandparent |  |
| Aunt/uncle |  |
| Sibling |  |
| Child carer |  |
| Other, please specify role: |  |

**Can I please check you’re all at least 16 years old?**

- 1. Provide a summary of the study:

Bathing babies can damage the skin and we want to find out whether advising families to bathe their babies less often and less intensively might stop babies having eczema. Before we can prove whether changing bathing practices might prevent eczema, we would like your help to discuss the challenges in asking families to change their baby bathing routine. We are planning a research study which will involve new mothers, and their families and child carers, being asked to bathe their new baby less intensively, and less frequently. By ‘bathing’ we mean bathing the whole body of your infant, and we would be asking new mothers to bathe their baby once a week or less and to use less skincare products meaning soaps, bubble bath etc, and also less moisturiser or oil, including oil for baby massage You could still wash your baby as much as you like – for example ‘topping and tailing’. We are interviewing you today to see what you think about this and, particularly, to hear whether you think there might be any difficulties with following this bathing routine.

- 1. The interview will last up to 45 minutes. The questions will generally be addressed to the mother to be, but you are all invited to comment.
  2. Each family will be offered a £40 retail voucher (“Love2Shop”).
  3. The interview will be audio recorded and anything you say will be kept anonymous, meaning your name will not be used in any reports.
  4. Have you any questions?

**Consent (if participant being interviewed remotely)**

Commence a recording specifically for the purposes of confirming the participants consent

Tell the participant that you are going to read out a series of statements from the consent form and that if they agree, after each one they should clearly say “I agree”

Read out each statement on the WP-1 consent form

Stop the recording and ensure it is saved

Complete a paper consent form confirming consent was taken and an audio recording made

**Warm up/context questions**

First, there are a few background questions I’d like to ask you (addressed just to mother):

How many weeks pregnant are you now? weeks

Do you have any children, if so, how many?

If they have children: do you mind telling me the name of your children, so that I can use their

names in our interview? Enter names, and refer to below:………………………………………………………………………

**General experience of and attitudes to infant bathing**

Now, I’d like to ask about your attitudes to bathing new babies.

If they have children:

What are your strongest memories of bath time with your child/children in the first weeks and months after they were born?

All

Do you have any memories of people in your family giving young children a bath, such as a younger sibling?

If they have children:

How often did xxxxxx/your child/children have a bath during the first weeks and months after they were born?

*Prompt*: *About how many times a week did they have a bath?*

How did you decide how often to bathe your baby?

*Prompt*: *Was it advice from: your midwife or health visitor, your family, reading about it, it’s what everyone you know does, just common sense, or same as I did with last baby?*

*If this hasn’t come up, ask “What advice did you receive from your midwife or health visitor, or NHS information (e.g., website) about how often to bathe your baby, in this pregnancy or previous pregnancies?”*

About how long did the baths last?

How warm were the baths and did you check if the bath was a suitable temperature, if so how?

Did you use products such as shampoo, oils, soap, or moisturiser in your baby’s bath, and do you give bubble baths, or do you use just water?

Did you use oils or moisturisers after the bath or at other times? How often were you moisturising or putting oil on the baby?

What about baby massage, as baby massage often involves using an oil or lotion. Did you do this? If so, how often?

Some people sometimes ‘top and tail’ their baby (i.e., just clean baby’s bottom and face). Did you do this? If yes, why did you decide to do this?

If you did “top and tail” your baby, did you see this as a substitute for a bath? Or something separate?

How do you think having other children will affect your bath time routine with the new baby?

*Prompt: If no effect, why not?*

All:

Have you thought about how often you will give your new baby a bath in the first weeks and months?

*Prompt*: *About how many times a week might you give your new baby a bath?*

How will you decide how often to give your new baby a bath?

*Prompt*: *Will it be advice from: your midwife or health visitor, your family, reading about it, it’s what everyone you know does, or just common sense?*

**Attitudes to bathing infant once a week or less**

*Adjust questions according to whether mother/family is planning to give the new baby a bath once a week or less or more than this.*

Next, I’d like to ask about your thoughts on the bathing routine we are planning.

What are your initial thoughts about this research, that would involve families being asked to give their new baby a bath once a week or less?

What do you see as the main advantages of bathing your baby once a week or less?

*Prompts: e.g., save time, more natural, convenience*

What do you see as the main disadvantages of bathing your baby once a week or less?

*Prompt: What might discourage you from continuing with this bathing routine?*

*Prompt*: *If baby sleeps better on a bathing day, if baby smells better on bath day, it is hygienic to bathe more frequently, it is an enjoyable experience to bathe your baby, part of bonding with your baby, miss bath time play/fun, judgement and opinions of others, looking forward to bathing baby*

How flexible are you about your plans for how often you would bathe the baby?

How confident do you feel that you could bathe your baby once a week or less, for at least six months, if asked to do so for a research study?

How likely would you be able to do this?

Is there a certain age when you would feel it is harder to follow such advice – for example when you might want to start to bathe your baby more frequently.

*Prompt* – *How would you feel about bathing your baby once a week or less once solid foods are introduced and feeding becomes quite messy? Would you be happy to just top and tail as necessary during this period? Do you see cleaning food away as part of a bathing routine or as something separate that takes place after eating?*

**Attitudes to using reducing the ‘intensity’ of bathing**

Next, I’d like to ask about your thoughts about reducing the intensity.

What are your initial thoughts about, for the first six months, just using soap if really necessary, not using bubble bath, or any product in the bathwater, avoiding moisturiser or massage oil and keeping the baths quite short?

Do you think there could be advantages to doing this?

*Prompts: e.g., save time, more natural, convenience*

Do you think there could be disadvantages to doing this?

*Prompt: What might discourage you from continuing with this bathing routine?*

*Prompt*: *dry skin, sleep routine, looking forward to bathing baby,*

How would you feel about bathing the baby at a temperature that we recommend, unless it’s a really hot day?

How confident do you feel that you could do this, for at least six months, if asked to do so for a research study?

How likely would you be able to do this?

If you were in this research study, are there things the research team could provide to support you to remember about the study and bathing your infant once a week or less with minimal intensity?

E.g., using a tick box on a calendar, a laminated card in bathroom, text messages, a simple app?

*Prompt: What might help you remember to bathe your baby once a week or less?*

*Prompt: If you were finding it difficult to bathe your baby only once a week, what would you do to help yourself get back on track?*

As part of the research, how would you feel about keeping a record of how often you are bathing your baby, topping and tailing, using skincare products such as creams, oils, moisturisers and bath products and undertaking baby massage?

What would be your preferred way to record this?

*Prompt*: *app, wall chart, paper diary, paper calendar.*

How frequently would you be prepared to do this?

*Prompt: e.g*. Would you be prepared to record once a week how frequently you bathed your infant and other aspects of your babies skincare over the last week?

Would you be happy to also record the temperature of the bath sometimes?

**Social influences**

Now I’d like to ask about the role and influence of your family and other people around you.

Is there anyone who couldn’t attend the interview today who might be involved in bathing your baby?

What do you think your family’s or child carer’s attitudes would be if you were to bathe your baby once a week or less?

*Prompts:*

*Do you think anyone you talk to about this with might not think it’s a good idea?*

*Do you think anyone you discuss this with might not approve?*

*How might this make you feel and how might you deal with their disapproval? - e.g. deal with it by explaining the reasons for bathing just once a week or less*

If you were taking part in a research study, how confident would you feel about explaining to your family and child carers that you are being asked to bathe your baby once a week or less?

How confident would you feel in convincing them to support you with this?

*Prompt*: *What would help you to convince them about the new routine? (e.g., reasons for doing it)*

If you were to explain to them that it’s part of a research study which may show that babies bathed once a week or less are less likely to develop eczema, would this make a difference?

*Prompt: Do you think they would accept this and be OK with it?*

How often do you think most people you know bathe a baby in the first few weeks and months after they are born?

How important do you think it is that you follow the same baby bathing routine as people you know?

*Prompt: same as your friends, or as people in your family?*

Do you know of any religious beliefs that might affect how often you bathe your baby?

*Prompt: if yes, how might these beliefs affect your bathing routine?*

**Baby’s skin health**

Now I’d like to ask about the health of a baby’s skin.

All

How do you think bathing might affect the health of a baby’s skin?

If your infant developed skin problems such as dry, red or sore skin, might this affect your bathing routine?

(e.g., bath less or more or about the same)

What about nappy rash? Would you feel comfortable just washing the face and bottom if your baby had nappy rash, or would you want to bathe the baby?

Can you tell me about any experience you have with yourself or anyone you know having eczema?

Prompt: Eczema is a skin condition that can make the skin red, dry and itchy.

Prompt: If the woman has had eczema – Has that affected how often you might want to bathe your baby?

How do you think bathing may influence an infant’s chances of developing eczema?

If they have children:

Have any of your children had eczema? If yes, did this affect your bathing routine?

All

What might you do to reduce the chances of your baby developing eczema? (.e.g, oils, creams, less bathing)

Do you think some babies are more likely to develop eczema? If so, why? (e.g., family history, ethnic background)

**Taking part in research trial**

Finally, I’d like to ask about what you would think if you were invited to take part in the planned research study.

How confident are you that you understand what the research would be asking families to do and the reasons for the research?

How important do you think the research is?

*Prompt*: *Might it be worthwhile bathing your baby once a week or less?*

If you were invited, would you be willing to take part in a research study testing how bathing babies once a week affects the health of baby’s skin?

If no, why wouldn’t you want to take part?

*Prompts*: *Too little information to decide, not interested in area of research, worried about intervention, views from family/friends etc.*

If Yes:

This would involve you having an equal chance of being given advice to support bathing your baby once a week or less often; or being given the standard St George’s newborn care advice.

How would you feel about joining the study?

Would you have any concerns about being given standard advice?

Who would you prefer to explain the intervention to bathe baby once a week or less as part of the research study?

*Prompt*: *a midwife, health visitor, practice nurse, researcher, don’t mind.*

When do you think would be the best stage in your pregnancy, or after your baby is born, for the research team to give you this advice about infant bathing and skin care?

*Prompt*: *Mid-pregnancy, late-pregnancy, early postpartum (e.g., on postnatal ward, health visitor appointment at 10-14 days after birth), combination.*

Why?

What are your thoughts about if an optimum frequency for baby bathing were demonstrated and this became a government guideline about how often to bathe your baby?

Manufacturers of bathing products for babies often try to influence the public by making suggestions about how often and how to bathe babies, what are your thoughts about this?

*Prompt: does thinking about this affect your thoughts about whether there should be a recommendation from the UK government about how often to bathe babies?*

Has any of this interview changed your views about how frequently you would bathe your baby?

*Prompt: might you bathe your baby less, more or about the same*

Has any of this interview changed your views about how intensively you would bathe your baby?

*Prompt: might you bathe your baby less intensively, more or about the same*

It’s important to us that the mix of people included in our research are representative of the UK population. Can I check how each of you would describe your ethnicity? *(tick and label with the role in the family)*

**Asian or Asian British**

- Indian
- Pakistani
- Bangladeshi
- Chinese
- Any other Asian background

**Black, Black British, Caribbean or African**

- Caribbean
- African
- Any other Black, Black British, or Caribbean background

**Mixed or multiple ethnic groups**

- White and Black Caribbean
- White and Black African
- White and Asian
- Any other Mixed or multiple ethnic background

**White**

- English, Welsh, Scottish, Northern Irish or British
- Irish
- Gypsy or Irish Traveller
- Roma
- Any other White background

**Other ethnic group**

- Arab
- Any other ethnic group (please complete) _____________________________

Have any of you ever been diagnosed with eczema by a doctor? (or ____, you told me about being diagnosed with eczema, has anyone else been diagnosed with eczema by a doctor?

Who? (role in family) _______________

Can you tell me your ages? (note here) _________________________________________________________

Highest education level of other interviewees _______________________________________________

**Assess interest in attending focus group (WP2) after their baby is born**

Would you be willing to take part in a focus group, after your baby is born, with other new mums, pregnant women and their families, to discuss the type of information we might provide to encourage women to bathe their babies less frequently? For example, this might involve you discussing information sheets we have prepared.

Yes  No

If interview is via telephone or videoconferencing, explain that the £40 Love2Shop vouchers will be posted or emailed to them.

Email address: _________________________________________________

Postal address:

|  |
| --- |
|  |

If interview is face-to-face, offer the £40 vouchers at the end of the interview

**Thank family for taking part**
