# Supplementary material for: Development of an intervention for reducing infant bathing frequency
Source: PLoS One. 2024 Feb 29;19(2):e0298335. doi: 10.1371/journal.pone.0298335 (PMC10903808; doi:10.1371/journal.pone.0298335)
Supplement: S3 File — (PDF) [file pone.0298335.s004.pdf]

BabyBathe Booklet

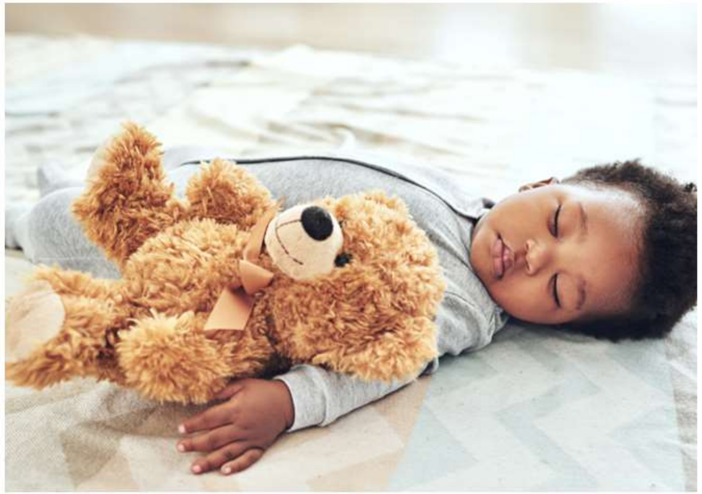

St George's University of London

Baby Bathe

NHS St George's University Hospitals NHS Foundation Trust

Thank you for joining BabyBathe!

Please aim to:

- **Bathe your baby once a week or less**
- **Keep baths short and not too hot**
- **Log when you bathe your baby on the MyCap app**

In this booklet you will find out about the study and things we are asking you to do, such as recording each time you bathe your baby in the study app.

Recap about the study and the science

Eczema is a skin condition that can make the skin red and itchy and one in five young children suffers with eczema. Eczema can cause stress for children and parents and affect a child's sleep. It often continues into adult life. There are many ways of treating childhood eczema, but we don't know how to prevent eczema.

Bathing babies can cause changes in their skin which might make eczema more likely to occur. Frequent bathing became common in Europe in the last century, which coincides with eczema becoming more common. We want to find out if families are willing to follow advice about infrequently bathing their baby, as this might help prevent eczema.

How was the study designed?

The study team includes a dermatologist, paediatricians, psychologists and an expert patient. We designed the study with advice from midwives, health visitors and GPs. We interviewed 20 families about our plans for the study and include quotes from these interviews in this booklet.

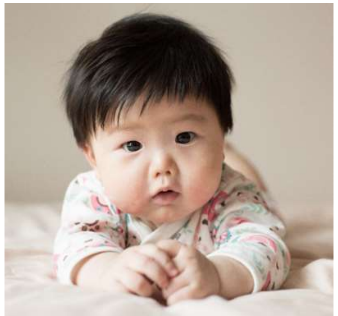

Taking part in the study

We would like you to:

- Bathe your baby once a week or less until your baby is six months of age.
- Record every time you bathe your baby using the study app.
- Complete monthly questionnaires. This should take less than 5 minutes.
- When your baby is 6 months old, we will invite you to bring your baby to St George's for a skin assessment and to complete a questionnaire. The visit will take 20-30 minutes.

Keeping in touch

We will contact you via email or the study app. Please let us know if you change your contact details. If we cannot contact you, we may contact the friend/relative you told us about, or your GP (unless you ask us not to), to check if you have changed contact details and/or to ask about your child's skin health.

What do we mean by bathing?

We mean immersing your baby in water (e.g., putting your baby in a bath or a baby bath). Wiping your baby's face and neck or bottom doesn't count as bathing.

Here's a mum commenting on bathing her baby once a week or less:

"It just seems to make logical sense. They're not doing anything and you're cleaning them every time you change their nappy, so where else are they getting dirty? As long as you're keeping them clean with nappy changes and little top and tail, like, strip wash."

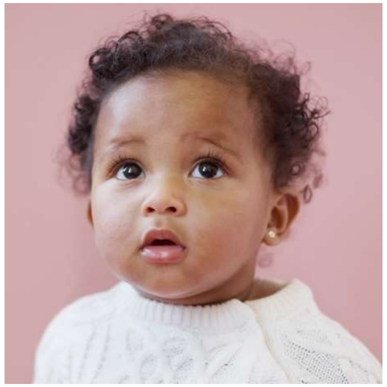

Keeping baths short and not too hot

To make baths easier on your baby's skin:

- Please keep baths short
- Plain water is sufficient in most instances
- You can use the thermometer provided in the pack to check the bath is not too hot

Topping and tailing

Topping refers to wiping your baby's face and tailing means wiping the nappy area, making sure to clean in all folds in the skin. This is used by many parents as a good alternative to bathing. You may want to use a bowl and cotton wool, or a flannel when they're older.

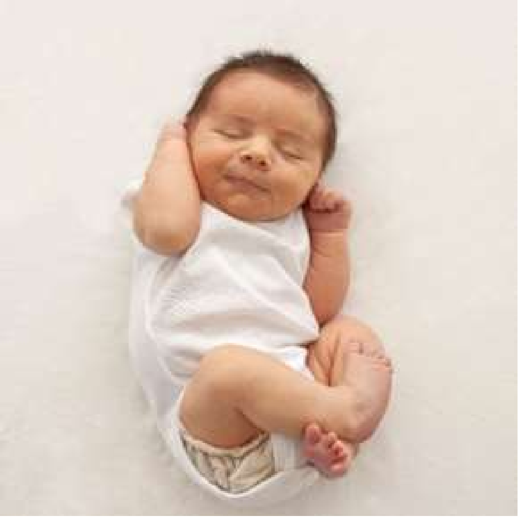

Establishing a baby bedtime routine

You may feel ready to establish a bedtime routine when your baby is around 3 months old. A simple, calming bedtime routine can be enjoyable for both you and your baby. Many parents find they don't need to include a bath in the bedtime routine. You might want to imagine what your nightly routine might be like. The routine could consist of:

- Changing into night clothes and a fresh nappy
- Dimming the lights to create a calm atmosphere
- Reading a bedtime story
- Singing a lullaby or having a musical mobile you can turn on when you've put your baby to bed
- Giving a goodnight kiss and cuddle
- Putting them to bed

Here's a mum talking about the nightly routine:

"I think maybe, instead of it being a bath, have the final nappy change with that little cleaning routine, and then they're into their PJs and then it's turn the lights down low, have a little night light, read a book, real chill, just try and do some sort of routines."

Using the MyCap study app

Please use the study app to tell us when you bathe your baby and to complete the monthly questionnaires. If you need to contact us, please do so via the app.

- When you join the study we will send you a personalised link. Click on the link to install the study app on you phone. You must allow notifications as we will use these to contact you.
- If a relative or carer is likely to be bathing the baby without you, please forward them the personalised link and ask them to install the app as you have done. Don't worry if you both tell us about the same bath as we will be able to remove duplicate entries. The data is collected centrally and you will not be able to see the data your partner, or your baby's grandparents entered.
- If you feel like you have to enter your passcode too often, you can turn this off in the app by selecting the menu on the top left, then 'passcode' and slide the blue button to 'off'
- We have a guide for the app which is available to download from the website (link and QR code on the back of the booklet)
- You may get a call from the study team to ask if you need help using the app.

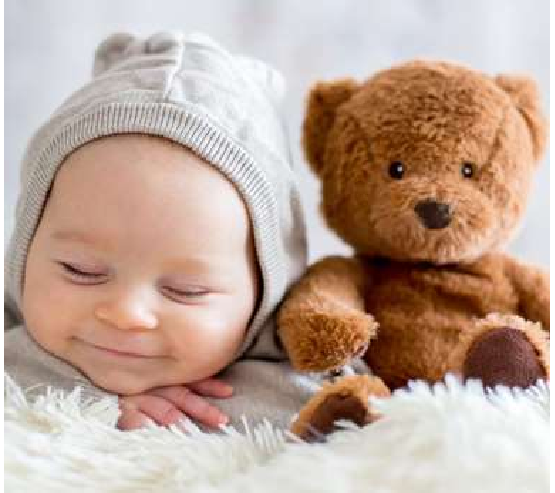

Using the study aids

We will post study aids to you make it easier for you take part in the study

To remember which day of the week you last bathed your baby on, you could:

- Use the plastic 'days of the week' bookmarks to attach to the shower curtain/screen to keep track of which day you last bathed your baby.

And/ or:

- Use the wallchart.

Please use the study reminders (fridge magnet and bookmark) to remind you and your loved ones who care for the baby about the study.

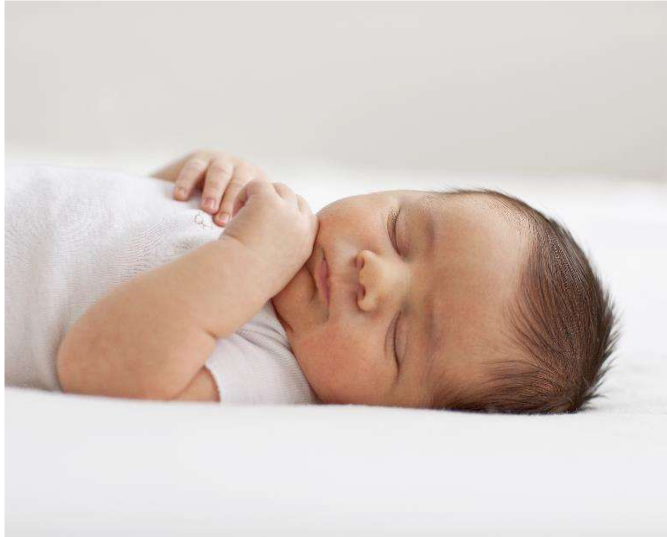

Frequently Asked Questions

Is it normal to only bathe a baby once a week or less?

The EAT study of 1,303 infants in England and Wales showed that one in six families bathe their baby once a week or less. In our interviews with 20 families expecting a baby, we found a large range in how often families were planning to bathe their baby – from every day to every 3 weeks.

Will people think it's strange that I only bathe my baby once a week or less?

Most people won't know how often you bathe your baby. You may choose to tell people about the study and let them know that 1 in 6 families in the UK bathe their baby once a week or less.

You can use this booklet to explain the study to your friends and family. You can talk about family experiences of eczema and how severe eczema can really affect some children, especially their sleep. You can talk about your participation in the study contributing towards science for the benefit of many.

"I think it is important to see if there is a causal link between the bathing and the eczema."

Are there hygiene concerns?

You can clean your baby's nappy area, or any sick, as normal. Your baby will not be smelly or dirty.

What if my baby develops a skin problem?

If your baby develops a skin problem, seek advice from a healthcare practitioner as you usually would. We ask about any skin problems in each monthly questionnaire that you will be completing.

Would bathing less affect bonding with my baby?

There are many ways to bond with your baby including skin-to-skin contact, cuddling, breastfeeding, gentle talk, singing and reading.

Why have other people joined the study?

Many people join research studies to contribute to science and to help others.

"The main question in my mind now is, does it actually bring on eczema in someone. I'd like to know earlier if you can just prevent it happening entirely. If that is the case, there's huge benefits."

How can my partner/family/caregivers help?

They can help in many ways:

- Encouraging and supporting each-other in ensuring your baby is bathed no more than once a week.
- Using the materials we provided to remember and record when you last bathed your baby.
- Helping with recording the bathing information on the app.

What if I'm finding it difficult to bathe the baby once a week or less?

If you are finding it difficult to bathe your baby no more than once a week **we very much want you to continue in the study**. The information you provide is just as important. Please continue to record your baby's bathing frequency in the study app and complete the questionnaires.

If and when you feel ready to try bathing your baby less frequently, aiming for once a week or less, please give it another go. Please don't blame yourself if you have difficulty following this. Remind yourself of when you found it easier to do and look for opportunities to get back on track!

Other benefits to being in the study

Here are some additional thoughts from families we have interviewed:

"We had to prepare a lot of stuff, we'd have to make sure the room was warm, the temperature's nice and then make sure that we dry them properly. And it is extra stuff that you have to do in your day, so the advantage of bathing less is easier on the mums, new parents."

"An environmental impact is probably less bathwater that's getting used. Less chemical products that are being used, less plastic bottles that are being used."

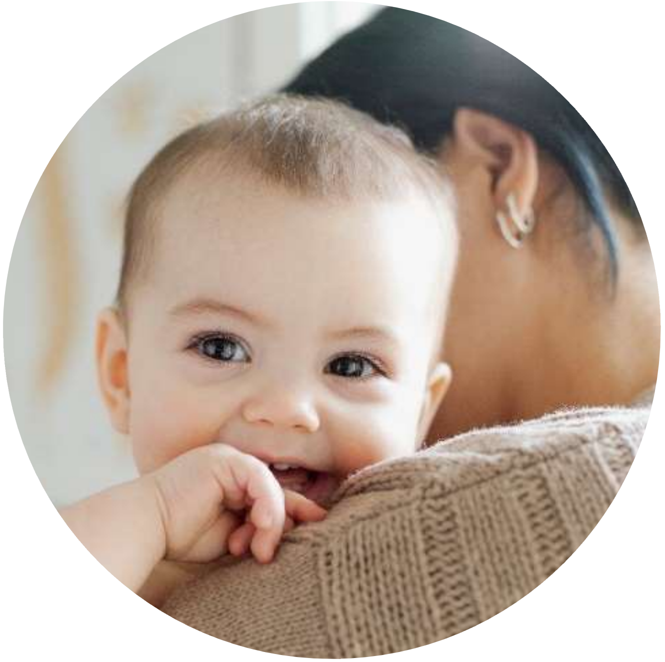

Contact Us

Please use the messaging feature in the study app to contact us. If this is difficult, you can email the study team at: babybathe@sgul.ac.uk

Online: use QR code or link
https://www.sgul.ac.uk/about/our-institutes/population-health/projects/babybathe/int
Twitter: @BabyBathe

St George's University of London

Baby Bathe

NHS
St George's University Hospitals
NHS Foundation Trust
