# Supplementary material for: Development of an intervention for reducing infant bathing frequency
Source: PLoS One. 2024 Feb 29;19(2):e0298335. doi: 10.1371/journal.pone.0298335 (PMC10903808; doi:10.1371/journal.pone.0298335)
Supplement: S4 File — (PDF) [file pone.0298335.s005.pdf]

BabyBathe Booklet

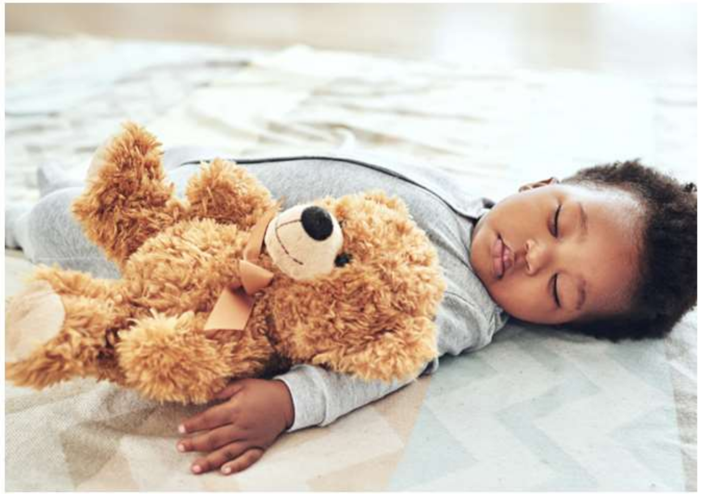

St George's University of London

Baby Bathe

NHS St George's University Hospitals NHS Foundation Trust

Thank you for joining BabyBathe!

Please log when you bathe your baby on the MyCap study app

In this booklet you will find out about the study and things we are asking you to do, such as recording each time you bathe your baby in the study app.

What do we mean by bathing?

We mean immersing your baby in water (e.g., putting your baby in a bath or a baby bath).

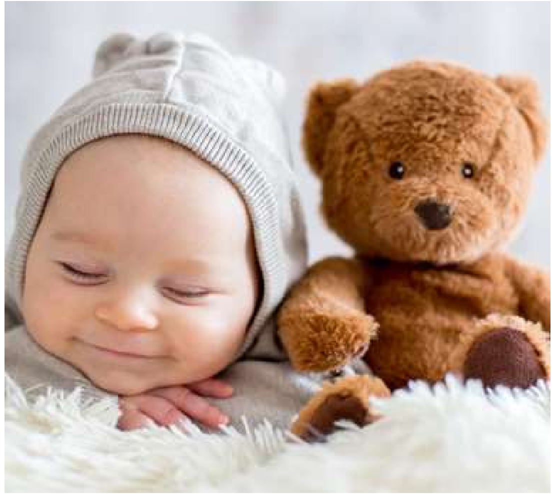

How was the study designed?

The study team includes a dermatologist, paediatricians, psychologists and an expert patient. We designed the study with advice from midwives, health visitors and GPs. We interviewed 20 families about our plans for the study.

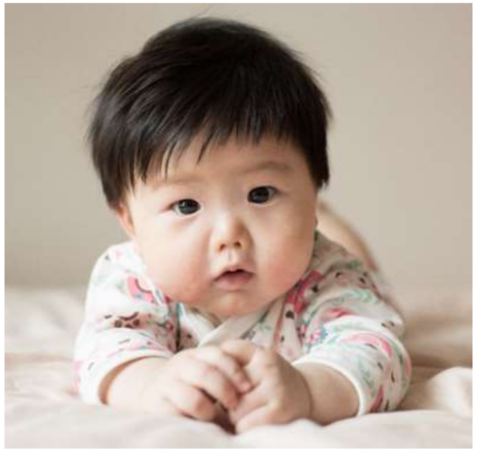

Taking part in the study

We would like you to:

- Record every time you bathe your baby using the study app.
- Complete monthly questionnaires. This should take less than 5 minutes.
- When your baby is 6 months old, we will invite you to bring your baby to St George's for a skin assessment and to complete a questionnaire. The visit will take 20-30 minutes.

Using the MyCap study app

Please use the study app to tell us when you bathe your baby and to complete the monthly questionnaires. If you need to contact us, please do so via the app.

- When you join the study, we will send you a personalised link. Click on the link to install the study app on your phone. You must allow notifications as we will use these to contact you.
- If a relative or carer is likely to be bathing the baby without you, please forward them the personalised link and ask them to install the app as you have done. Don't worry if you both tell us about the same bath as we will be able to remove duplicate entries. The data is collected centrally and you will not be able to see the data your partner, or your baby's grandparents entered.
- If you feel like you have to enter your passcode too often, you can turn this off in the app by selecting the menu on the top left, then 'passcode' and slide the blue button to 'off'
- We have a guide for the app which is available to download from the website (link and QR code on the back of the booklet)
- You may get a call from the study team to ask if you need help using the app.

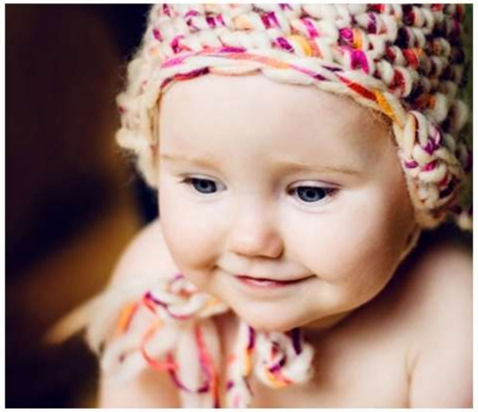

Keeping in touch

We will contact you via email or the study app. Please let us know if you change your contact details. If we cannot contact you, we may contact the friend/relative you told us about, or your GP (unless you ask us not to), to check if you have changed contact details and/or to ask about your child's skin health.

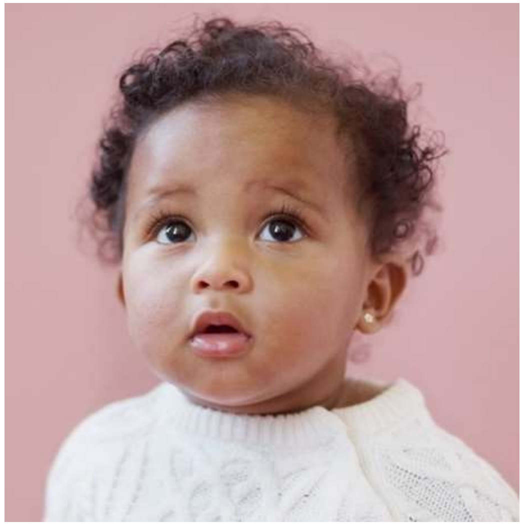

Frequently Asked Questions

What if my baby develops a skin problem?

If your baby develops a skin problem, seek advice from a healthcare practitioner as you usually would. We ask about any skin problems in each monthly questionnaire that you will be completing.

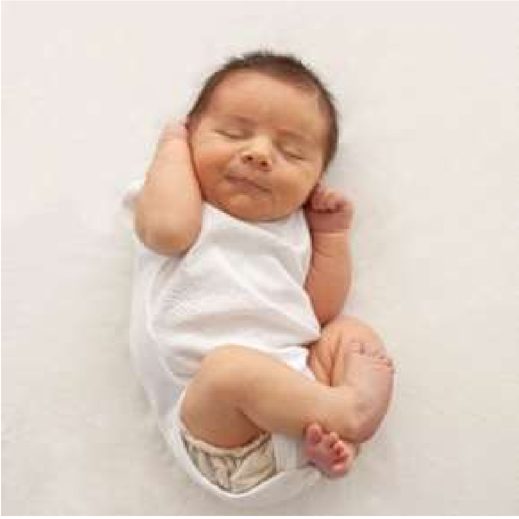

How do I explain the study to my friends and family?

You can use this booklet to explain the study to your family friends. You may want to talk about the study contributing towards science for the benefit of many.

How can my partner/family/caregivers help?

They can help in many ways:

- Encouraging and supporting each other to remember to record when you bathe your baby and to help recording bathing information in the study app.

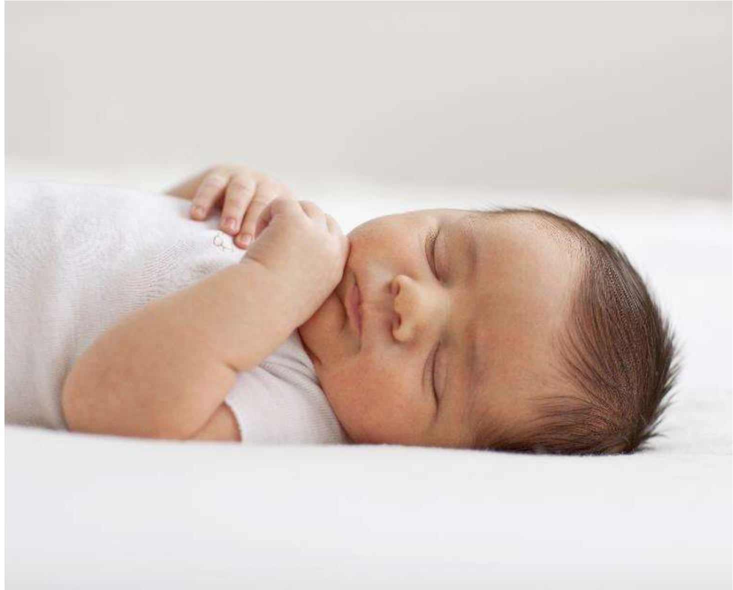

Why have other people joined the study?

Many people join research studies to contribute to science and to help others.

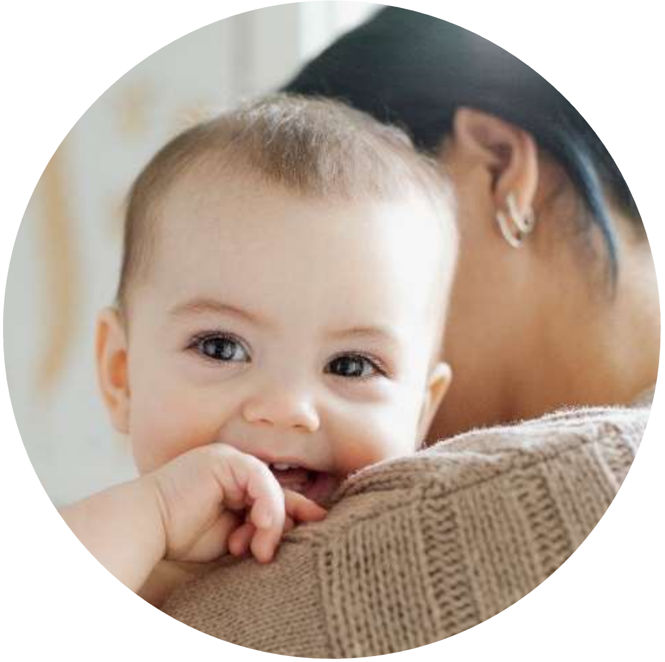

Contact Us

Please use the messaging feature in the study app to contact us. If this is difficult, you can email the study team at:
babybathe@sgul.ac.uk

Online: use QR code or link:
https://www.sgul.ac.uk/about/our-institutes/population-health/projects/babybathe/cont
Twitter: @BabyBathe

St George's University of London

Baby Bathe

NHS St George's University Hospitals NHS Foundation Trust
