# Supplementary figures and images for: Development of an intervention for reducing infant bathing frequency
Source: PLoS One. 2024 Feb 29;19(2):e0298335. doi: 10.1371/journal.pone.0298335 (PMC10903808; doi:10.1371/journal.pone.0298335)

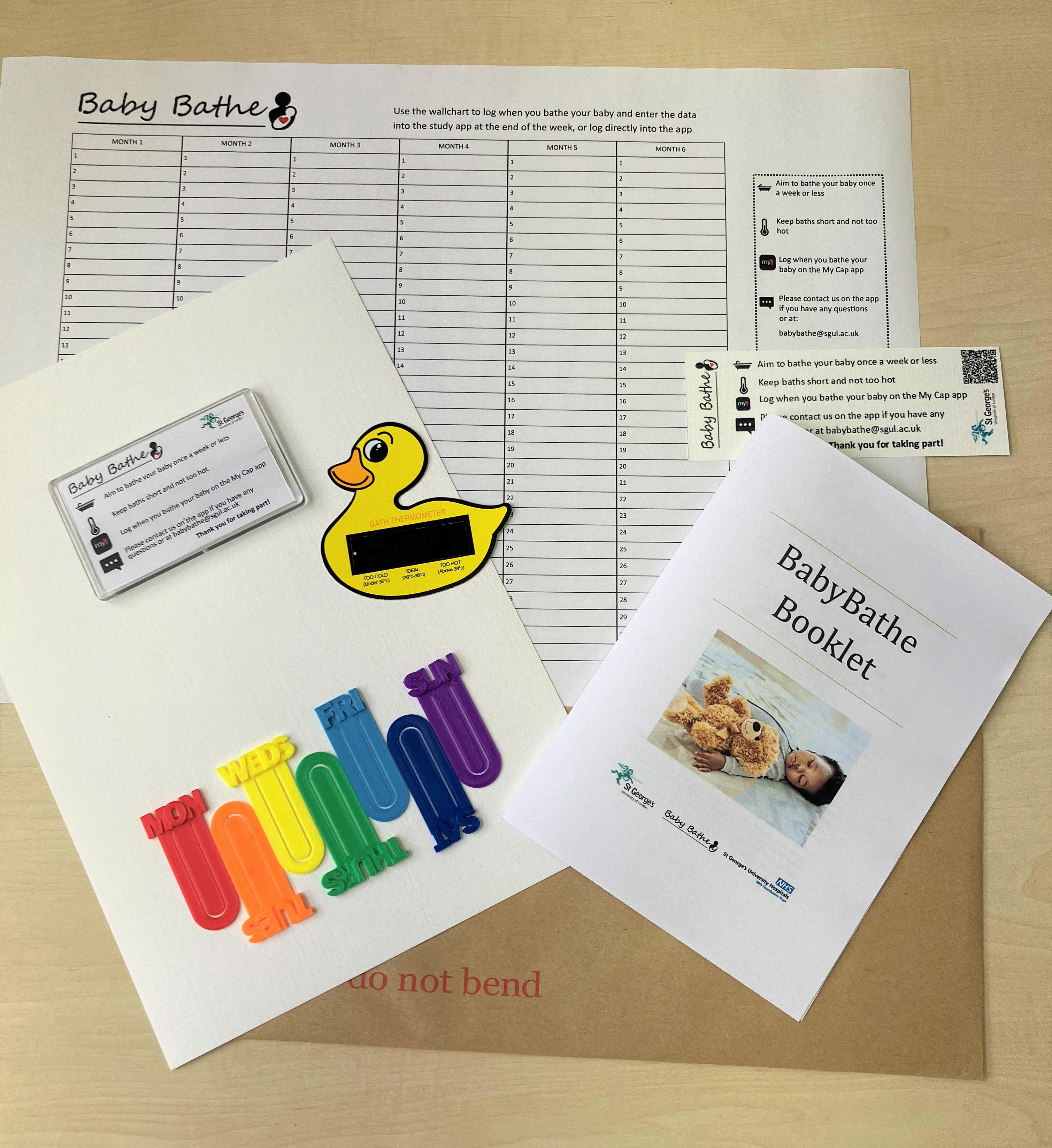

Supplement: S1 Fig — (JPEG) [file pone.0298335.s006.jpeg]

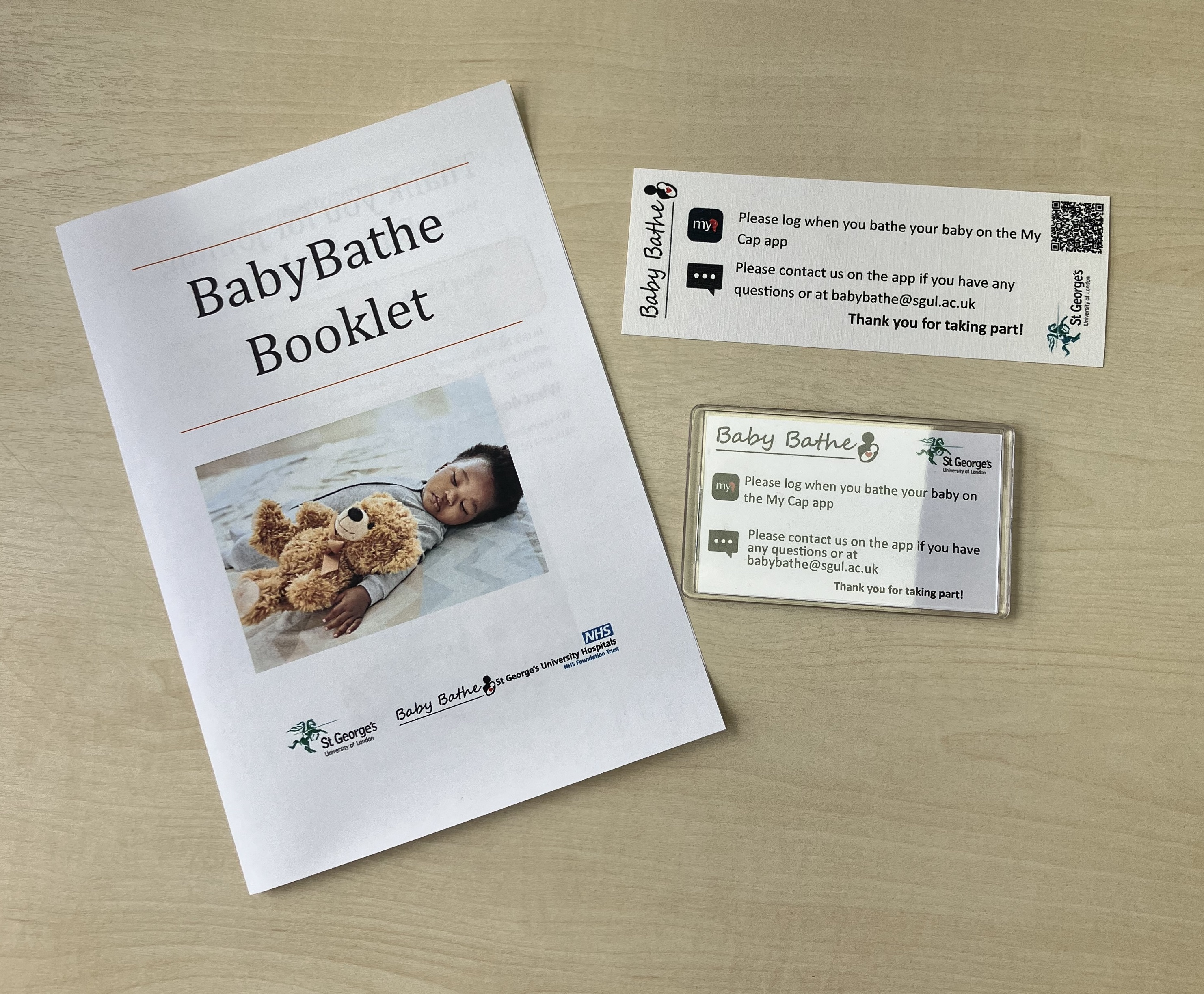

Supplement: S2 Fig — (JPG) [file pone.0298335.s007.jpg]
