# Supplementary material for: Development of an intervention for reducing infant bathing frequency
Source: PLoS One. 2024 Feb 29;19(2):e0298335. doi: 10.1371/journal.pone.0298335 (PMC10903808; doi:10.1371/journal.pone.0298335)
Supplement: Attachment — Submitted filename: Response to reviewers.docx [file pone.0298335.s008.docx]

**Response to editors and reviewers**

**Editor’s comments**

1. Please ensure that your manuscript meets PLOS ONE's style requirements, including those for file naming. The PLOS ONE style templates can be found at: https://journals.plos.org/plosone/s/file?id=wjVg/PLOSOne_formatting_sample_main_body.pdf and 
https://journals.plos.org/plosone/s/file?id=ba62/PLOSOne_formatting_sample_title_authors_affiliations.pdf

**RESPONSE**: We have revised the manuscript to follow PLOS ONE’s style requirements, including requirements for font size and headings and ordering of sections. These changes are shown in the tracked version of the manuscript. Our file names adhere to PLOS ONE’s requirements.

2. Please note that in order to use the direct billing option the corresponding author must be affiliated with the chosen institute. Please either amend your manuscript to change the affiliation or corresponding author, or email us at plosone@plos.org with a request to remove this option.

**RESPONSE**: We have changed the corresponding author from Lucy Goldsmith to Michael Ussher. This because Lucy has now left St George’s, University of London and to take advantage of the institutional partnership between PLOS ONE and St George's we need to have a corresponding author at St George’s.

3. In your Data Availability statement, you have not specified where the minimal data set underlying the results described in your manuscript can be found. PLOS defines a study's minimal data set as the underlying data used to reach the conclusions drawn in the manuscript and any additional data required to replicate the reported study findings in their entirety. All PLOS journals require that the minimal data set be made fully available. For more information about our data policy, please see http://journals.plos.org/plosone/s/data-availability.

Upon re-submitting your revised manuscript, please upload your study’s minimal underlying data set as either Supporting Information files or to a stable, public repository and include the relevant URLs, DOIs, or accession numbers within your revised cover letter. For a list of acceptable repositories, please see http://journals.plos.org/plosone/s/data-availability#loc-recommended-repositories. Any potentially identifying patient information must be fully anonymized.

Important: If there are ethical or legal restrictions to sharing your data publicly, please explain these restrictions in detail. Please see our guidelines for more information on what we consider unacceptable restrictions to publicly sharing data: http://journals.plos.org/plosone/s/data-availability#loc-unacceptable-data-access-restrictions. Note that it is not acceptable for the authors to be the sole named individuals responsible for ensuring data access.

We will update your Data Availability statement to reflect the information you provide in your cover letter.

**RESPONSE**: We have updated our data availability statement as follows:

“Most of the relevant data are within the Results section of the manuscript, in the form of excerpts of the transcripts. Additional data, we refer to in the manuscript, is available in an online repository, in the form of an interactive mapping diagram, illustrating connections between different elements of the data: https://doi.org/10.24376/rd.sgul.23966574”

(see Manuscript, p. 23, Final Intervention section)

4. Please review your reference list to ensure that it is complete and correct. If you have cited papers that have been retracted, please include the rationale for doing so in the manuscript text, or remove these references and replace them with relevant current references. Any changes to the reference list should be mentioned in the rebuttal letter that accompanies your revised manuscript. If you need to cite a retracted article, indicate the article’s retracted status in the References list and also include a citation and full reference for the retraction notice.

**RESPONSE**: We have reviewed the reference list, which was originally submitted, and can confirm that it is complete and correct.

**Reviewer’s Comments**
Reviewer #1

**COMMENT:** The article's proposal, entitled "Development of an intervention for reducing infant bathing frequency", is very well presented and corresponds to public health research with scientific relevance, with a concrete and realistic question that can have practical and effective application in health prevention.

**RESPONSE**: Thank you for these positive comments.

**COMMENT:** The project was duly approved by an ethical opinion issued by the North of Scotland Research Ethics Committee. I want to ask why this was done with a committee in Scotland and not with a committee in London or St George's Hospital, London.

**RESPONSE**: We went to the ethics board with first availability to review our study proposal. This happened to be the North of Scotland Ethics Board. The process was an online submission and online interview, so the distance did not matter.

**COMMENT:** The authors adequately present the Consolidated Criteria for Reporting Qualitative Research (COREQ) items. The methodology is well structured, clearly presenting the inclusion criteria and intervention steps to recognize the likely positive and negative factors influencing acceptance to participate in an evaluation study. Interestingly, the authors state, "Our epistemological stance in this study was critical realism – which assumes that experiences are understood through human interpretation and mediated by our beliefs and perceptions”.
The results show a precise sequence of stages and the inclusion of feedback in developing the intervention materials. Indeed, as the authors rightly state, the intervention design process incorporated the "real world" views and experiences of families, experts, and PPIE representatives.

**RESPONSE**: Again, thank you for this positive appraisal.

**COMMENT:** The abstract's conclusions do not state a significant result for behaviour change techniques: education, persuasion, and restructuring of the environment. These techniques should be included in the abstract's conclusions.

**RESPONSE:** We thank you noting this omission. We have added the following text to the Results section of the abstract. We think it fits more appropriately in the Results section than in the Conclusion.

“…focussing on the intervention types of education, persuasion and environmental restructuring…’
